# When anaphylactic shock meets epinephrine and blood lactate increases: A case report

**DOI:** 10.1097/MD.0000000000039263

**Published:** 2024-09-06

**Authors:** Zhi-Rong Zhang, Ying-Ying Shen, Ru-Qin Yi, Wen Feng, Wei Chen, Cheng-En Li, Zhao-Kun Fan

**Affiliations:** a Department of Intensive Care Unit, The First Affiliated Hospital of Zhejiang Chinese Medical University (Zhejiang Provincial Hospital of Chinese Medicine), Hangzhou, China; b Department of Medical Record, The First Affiliated Hospital of Zhejiang Chinese Medical University (Zhejiang Provincial Hospital of Chinese Medicine), Hangzhou, China.

**Keywords:** anaphylactic shock, blood lactate, epinephrine

## Abstract

**Rationale:**

Anaphylactic shock, a severe and rapid systemic allergic reaction, poses significant treatment challenges. Epinephrine, the first-line treatment, effectively reverses symptoms but can complicate the clinical picture by elevating lactate levels, blurring the distinction between shock-induced hypoperfusion and drug-induced metabolic effects.

**Patient concerns:**

A 26-year-old female presented with anaphylactic shock following an antibiotic infusion, experiencing chest tightness, hypotension, and pulmonary edema, without significant past medical history apart from a noted allergy to fish and shrimp.

**Diagnoses:**

Anaphylaxis was diagnosed based on clinical presentation and supported by imaging that revealed pulmonary edema, despite normal troponin levels and electrocardiogram.

**Interventions:**

Treatment included 0.5 mg of intramuscular epinephrine and 5 mg of intravenous dexamethasone, with subsequent intubation and mechanical ventilation in the intensive care unit. An intravenous epinephrine infusion was also administered for hemodynamic support.

**Outcomes:**

While epinephrine resolved the pulmonary edema and stabilized circulation, it led to a significant, albeit transient, increase in lactate levels, which normalized following discontinuation of epinephrine, indicating the metabolic effect of the drug rather than ongoing tissue hypoperfusion.

**Lessons:**

This case illustrates the importance of recognizing epinephrine-induced lactate elevation in anaphylactic shock, necessitating a nuanced interpretation of lactate dynamics. Clinicians must differentiate between lactate elevations due to tissue hypoperfusion and those arising from epinephrine’s pharmacologic effects to optimize patient care.

## 1. Introduction

Anaphylactic shock represents an acute, severe systemic allergic reaction, characterized by rapid development and potential life-threatening consequences, commonly triggered by allergies to food, medications, or insect stings.^[1]^ The first-line treatment for anaphylactic shock is epinephrine due to its capacity to quickly reverse the symptoms of shock, including vasodilation, hypotension, and airway constriction.^[2]^ Epinephrine acts by stimulating α- and β-adrenergic receptors, increasing cardiac output, and raising peripheral vascular resistance, thereby improving tissue perfusion.

However, the use of epinephrine in emergency treatment, especially with sustained or repeated administration, has been observed to sometimes result in elevated blood lactate levels. Elevated blood lactate is typically considered a marker of tissue hypoxia, but, in the context of epinephrine treatment, the underlying mechanisms may be more complex. Epinephrine increases glycolysis, leading to enhanced anaerobic metabolism, which can, in turn, lead to increased production of lactate. In addition, epinephrine may affect lactate levels through other mechanisms, such as increasing muscle activity or directly influencing lactate metabolism.

Although this phenomenon has been noted, further research is needed to understand the specific mechanisms, clinical significance, and management of elevated blood lactate levels induced by epinephrine. This report aims to explore the relationship between the use of epinephrine and elevated blood lactate levels through the analysis of a case involving a patient with anaphylactic shock and how to identify and manage this phenomenon in clinical practice.

## 2. Case report

**Figure 1.**
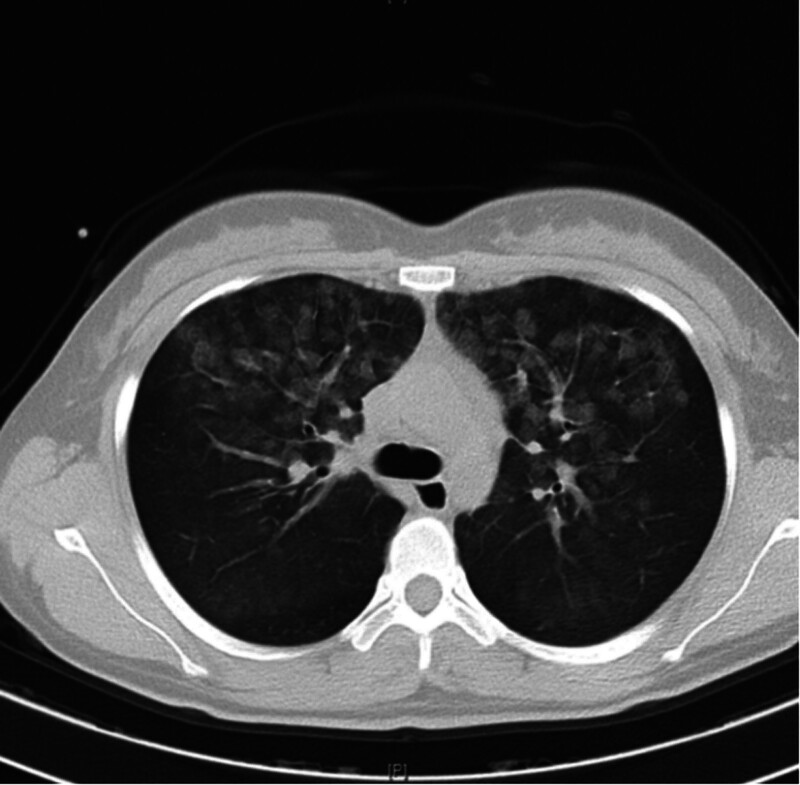
Diffuse pulmonary edema in both lungs.

A 26-year-old female with no significant past medical history, other than a noted allergy to fish and shrimp, presented to the emergency department with complaints of a rash and chest tightness that began 2 hours following an infusion of clindamycin and cefmetazole. Upon examination, she exhibited symptoms indicative of an acute allergic reaction, including chest tightness, a reduced oxygen saturation of 90%, hypotension (87/59 mm Hg), and audible rales upon auscultation of the lungs. Diagnostic tests, including troponin levels (Table 1) and electrocardiogram, showed no abnormalities, yet a chest computed tomography scan revealed evidence of pulmonary edema (Fig. 1). Based on these clinical findings, anaphylaxis was considered the primary diagnosis.

**Table 1:** Laboratory testing tends.

Variable	Reference range, adults	On arrival, this hospital	On arrival at ICU, 2 h after arrival, this hospital	On arrival at ICU, 1 d after arrival, this hospital
Hemoglobin, g/dL	11.5–15.0	13.6	12.8	12.6
Platelet count/μL	125,000–350,000	221,000	257,000	192,000
White cell count/μL	3500–9500	11,900	21,000	20,000
Sodium, mmol/L	137–147	138	140	141
Potassium, mmol/L	3.5–5.3	3.0	3.2	3.9
Creatinine, µmol/L	45–84	74	62	38
Glucose, mmol/L	3.89–6.11	10.37	13.3	7.3
Total protein, g/dL	6.5–8.5	6.4	5.7	5.8
Albumin, g/dL	4.0–5.5	4.4	4.0	4.1
Total bilirubin, µmol/L	3.4–20.5	10.4	17.5	13.7
Prothrombin time, s	9.8–14.0	12.4	12.8	12.2
Partial‑thromboplastin time, s	25.0–36.0	26.6	26.1	25.6
Troponin T, µg/L	0.00–0.04	0.04		2.60
Creatine kinase, U/L	40–200	28	100	11,012
Creatine kinase MB isoenzyme, U/L	0.0–24.0	1.0	23	552.0

ICU indicates intensive care unit.

Immediate treatment was initiated with 0.5 mg of intramuscular epinephrine and 5 mg of intravenous dexamethasone, alongside oxygen supplementation via a nasal cannula and fluid resuscitation. Subsequent to these interventions, the patient was transferred to the intensive care unit for further management.

Approximately 2 hours after admission to the intensive care unit, the patient’s condition deteriorated, characterized by decreased oxygenation, drowsiness, and the production of blood-tinged sputum. Arterial blood gas analysis revealed an oxygenation index of 100 and a lactate level of 4.8 mmol/L, indicating significant metabolic stress. Due to worsening pulmonary edema, she was intubated and placed on mechanical ventilation with adjusted positive end-expiratory pressure settings. In addition, an intravenous infusion of 2 mg adrenaline diluted in 50-mL saline, with an initial rate of 4 mL/h, up to a maximum of 6 mL/h, adjusted based on continuous monitoring of blood pressure and heart rate.

**Figure 2.**
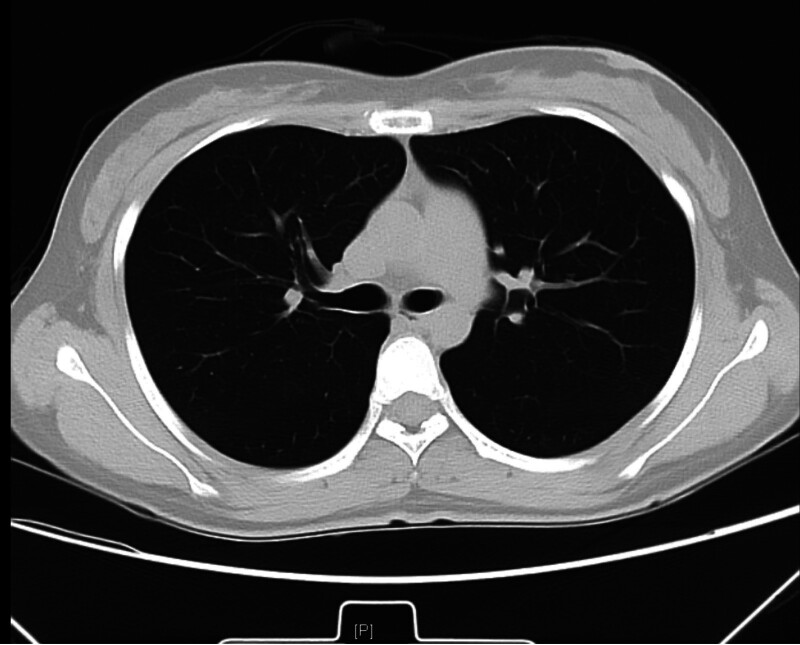
Pulmonary edema resolves.

**Figure 3.**
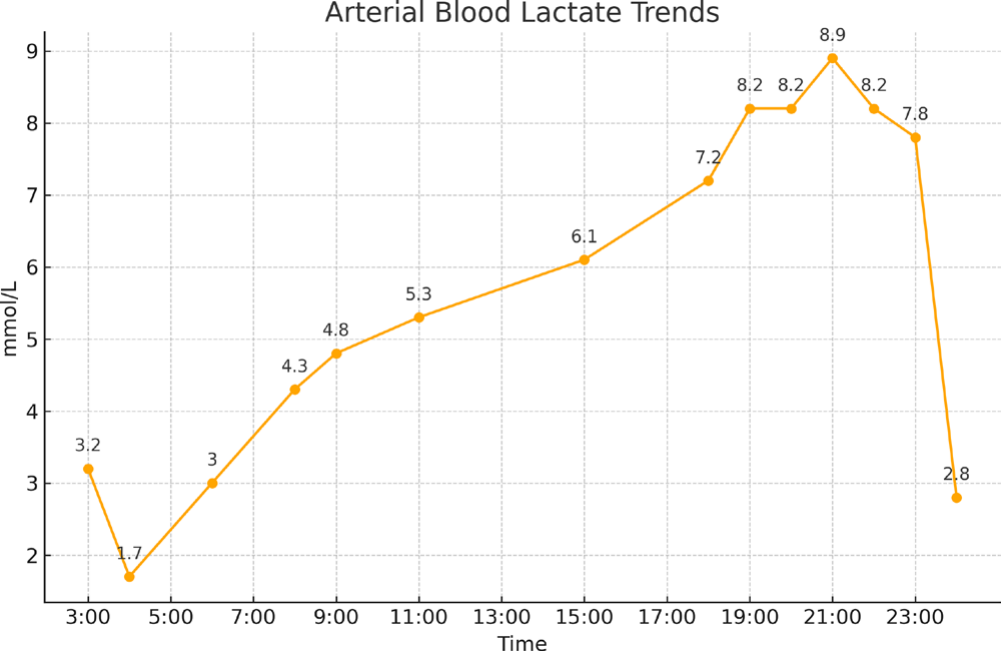
Lactic acid tends.

Following these interventions, the patient’s respiratory and hemodynamic status notably improved. Bedside ultrasound revealed a left ventricular outflow tract velocity time integral of 24 cm, indicating no significant decrease in cardiac output; the previously observed bloody, foamy sputum was no longer present, oxygenation levels gradually increased, lung rales resolved (Fig. 2), and circulatory stability was achieved. However, despite these clinical improvements and stable circulation, serial blood gas analyses demonstrated a gradual increase in lactate levels (Fig. 3), suggesting ongoing metabolic stress or altered lactate metabolism despite the resolution of acute symptoms and stabilization of hemodynamics.

Given the stabilization of the patient’s oxygenation and circulation, her lactate levels normalized shortly after discontinuation of epinephrine.

## 3. Discussion

The observation of elevated lactate levels in a young female patient following treatment for anaphylactic shock necessitates a detailed exploration to understand the pathophysiological mechanisms at play, interpret the clinical data accurately, and extract insights for guiding future therapeutic strategies. Elevated blood lactate, indicative of metabolic dysregulation, requires a nuanced analysis to ascertain the contributing factors and their implications for patient management in acute care settings.

### 3.1. Pathophysiology of elevated lactate

The accumulation of lactate in the bloodstream is a multifaceted process, potentially stemming from an upsurge in lactate production, a decrement in its utilization, or a combination thereof. Specifically, 3 principal mechanisms are implicated in the pathogenesis of lactate elevation^[3]^: enhanced production of pyruvate, which under aerobic conditions, is preferentially converted to lactic acid in the presence of NADH and H+, culminating in an elevation of lactate concentrations^[4]^; impeded translocation of pyruvate into the mitochondria, thereby hindering its oxidation to carbon dioxide and water or its conversion to glucose precursors, contributing further to the lactate pool; and a shift in the cytoplasmic redox balance toward a state characterized by an abundance of NADH relative to NAD+, favoring the conversion of pyruvate to lactate.

Lactic acidosis can be classified into 2 types. Type-A lactic acidosis is caused by hypoperfusion and hypoxia, such as in various shock states. Type-B lactic acidosis is defined as not involving tissue hypoxia or hypoperfusion. While type-B lactic acidosis may be less common than type-A, both share the fundamental problem of the mitochondria being unable to process the amount of pyruvate they receive. Consequently, alternative metabolic pathways for pyruvate described in the lactic acid cycle are activated, leading to elevated lactate levels. Examples of type-B lactic acidosis include liver disease, malignancy, medications (such as metformin and epinephrine), total parenteral nutrition, human immunodeficiency virus, thiamin deficiency, mitochondrial myopathy, congenital lactic acidosis, trauma, excessive exercise, diabetic ketoacidosis, and ethanol intoxication.^[5,6]^

### 3.2. Clinical interpretation of elevated lactate

The diagnostic evaluation of increased lactate levels in this particular case revealed several pertinent findings. The absence of tissue hypoperfusion, as evidenced by stable blood pressure, satisfactory urine output, central venous oxygen saturation exceeding 65%, and no significant decrease in cardiac output was observed as evaluated by bedside ultrasound, effectively excluded increased anaerobic metabolism due to inadequate tissue oxygenation as a causative factor. Likewise, the stability of coagulation profiles alongside liver and renal function tests preepisode and postepisode negated the likelihood of impaired lactate clearance attributable to organ dysfunction. Consequently, the adrenergically mediated acceleration of glycolysis and lipolysis,^[7]^ induced by epinephrine administration, emerges as the most plausible explanation for the observed hyperlactatemia. This pathway enhances pyruvate and free fatty acid levels, with the latter inhibiting pyruvate dehydrogenase activity, thereby retarding the conversion of pyruvate to acetyl-CoA and precipitating an accumulation of lactate.^[8,9]^

The delineation of these mechanisms highlights the intricate interplay between pharmacological interventions, such as epinephrine in the treatment of anaphylactic shock, and their metabolic repercussions. Understanding these interactions is pivotal for the comprehensive management of patients, enabling clinicians to mitigate potential adverse effects while maximizing therapeutic efficacy.

### 3.3. Clinical tips

#### 3.3.1. Contextual interpretation of hyperlactatemia

It is crucial to interpret elevated lactate levels within the context of the patient’s specific clinical scenario, including their medical history and response to treatment, rather than in isolation.

#### 3.3.2. Beyond inadequate perfusion

While inadequate perfusion is a common consideration in differential diagnosis, it is important to recognize that hyperlactatemia can also result from the pharmacological effects of certain drugs on the body, such as linezolid and metformin, which are known to potentially affect lactate levels in the body, and may not always correlate with the severity of the disease process.

#### 3.3.3. Epinephrine in anaphylaxis

As the first-line treatment for anaphylaxis, the metabolic effects of epinephrine on the body must be closely monitored. This includes understanding its relationship with adverse outcomes, such as hyperlactatemia, and managing these effects alongside the primary treatment goals.

## 4. Conclusion

This case underscores the complex interplay between epinephrine administration in anaphylactic shock and subsequent metabolic effects, including lactate elevation, highlighting the need for careful monitoring and management of these patients beyond the immediate resolution of anaphylactic symptoms.

## Author contributions

**Data curation:** Zhao-Kun Fan.

**Writing – review & editing:** Zhi-Rong Zhang.

**Software:** Ying-Ying Shen.

**Visualization:** Ru-Qin Yi.

**Formal analysis:** Wen Feng, Wei Chen.

**Investigation:** Wei Chen.

**Writing – original draft:** Cheng-En Li.
